# Th17 cells favor inflammatory responses while inhibiting type I collagen deposition by dermal fibroblasts: differential effects in healthy and systemic sclerosis fibroblasts

**DOI:** 10.1186/ar4334

**Published:** 2013-10-10

**Authors:** Nicolò Costantino Brembilla, Elisa Montanari, Marie-Elise Truchetet, Elena Raschi, Pierluigi Meroni, Carlo Chizzolini

**Affiliations:** 1 Immunology and Allergy, University Hospital and School of Medicine, Rue Gabrielle Perret-Gentil 4, Geneva 14, 1211, Switzerland; 2 Experimental Laboratory of Immunological and Rheumatologic Researches, IRCSS Istituto Auxologico Italiano, Milan, Italy; 3 Division of Rheumatology, Istituto G Pini, Department of Clinical Sciences and Community Health, University of Milan, Milan, Italy

## Abstract

**Introduction:**

T helper (Th)-17 cells are increased in systemic sclerosis (SSc). We therefore assessed whether Th17 cells could modulate the inflammatory and fibrotic responses in dermal fibroblasts from healthy donors (HD) and SSc individuals.

**Methods:**

Fibroblasts were obtained from 14 SSc and 8 HD skin biopsies. Th17 clones were generated from healthy peripheral blood upon enrichment of CC chemokine receptor (CCR)-4/CCR6/CD161 expressing cells. Their cytokine production was assessed by flow cytometry and multiplex beads immunoassay. Fibroblast production of monocyte chemoattractant protein (MCP)-1, interleukin (IL)-8, matrix metalloproteinase (MMP)-1, tissue inhibitor of metalloproteinase (TIMP)-1, MMP-2 and type-I collagen was quantified by enzyme-linked immunosorbent assay (ELISA) and radioimmunoassay (RIA), and changes in their transcription levels assessed by real-time PCR. Intracellular signals were dissected by western blot and the use of pharmacological inhibitors. IL-17A, tumor necrosis factor (TNF) and interferon-gamma (IFN-γ) blocking reagents were used to assess the specificity of the observed effects.

**Results:**

IL-17A increased MCP-1, IL-8 and MMP-1 production in a dose-dependent manner while having no effect on type I collagen in HD and SSc fibroblasts both at protein and mRNA levels. Nuclear factor-kappa B (NF-κB) and p38 were preferentially involved in the induction of MCP-1 and IL-8, while MMP-1 was most dependent on c-Jun N-terminal kinase (JNK). Supernatants of activated Th17 clones largely enhanced MCP-1, IL-8 and MMP-1 while strongly inhibiting collagen production. Of note, the production of MCP-1 and IL-8 was higher, while collagen inhibition was lower in SSc compared to HD fibroblasts. The Th17 clone supernatant effects were mostly dependent on additive/synergistic activities between IL-17A, TNF and in part IFN-γ. Importantly, the inhibition of type I collagen production induced by the Th17 clone supernatants was completely abrogated by blockade of IL-17A, TNF and IFN-γ mostly in SSc fibroblasts, revealing an intrinsic resistance to inhibitory signals in SSc.

**Conclusions:**

Our findings demonstrate that *in vitro* Th17 cells elicit pro-inflammatory responses while restraining collagen production. Thus, the increased Th17 cell number observed in SSc may impact on the inflammatory component of the disease simultaneously potentially providing a protective role against fibrosis.

## Introduction

Systemic sclerosis (SSc) is an autoimmune disorder of unknown origin characterized by fibro-proliferative microangiopathy and progressive fibrosis of the skin and internal organs [1,2]. Fibrosis results from an overproduction of extracellular matrix (ECM) components by fibroblasts, especially type I collagen, accompanied by impaired ECM degradation. In early SSc, dermal fibroblasts display an inappropriate phenotype essentially characterized by increased proliferative potential, increased synthetic capacity, resistance to inhibitory signals and decreased apoptosis [1,2]. In addition to collagens and matrix metalloproteinases (MMP), fibroblasts release several pro-inflammatory chemokines, such as monocytes chemoattractant protein (MCP)-1 and interleukin (IL)-8, which may indirectly influence ECM remodeling [3]. Of interest, MCP-1 and IL-8 are increased in the skin and serum of SSc patients [4,5] and appear to be critical in mediating bleomycin-induced lung and dermal fibrosis [6,7].

The mechanisms leading to dysregulated activation of fibroblasts in SSc are only partially understood. T cells infiltrate SSc skin early and fibroblasts with high synthetic activity localize in close proximity to the inflammatory infiltrate (reviewed in [8]). T helper (Th) 2 polarized responses have been shown to be dominant in SSc skin and lung [9-13]. Consistently, IL-4 and IL-13 were shown to have direct pro-fibrotic activities on fibroblasts both *in vitro* and *in vivo*[14]. In addition, we and others have reported that SSc individuals have increased Th17 cell counts in their peripheral blood and skin [15-21].

Th17 cells are physiologically implicated in protection against extracellular bacteria and fungi [22] and are thought to have pathogenic roles in various autoimmune diseases [23-25]. Th17 cells mainly produce IL-17A, in conjunction with IL-17 F, IL-21 and IL-22, and are enriched in the subset of T cells expressing the chemokine receptors CCR4 and CCR6 in the absence of CCR10 [26,27]. They further express the lectin receptor CD161 [28]. IL-17A has been shown to participate in the development of skin and lung fibrosis induced by bleomycin in mice [29,30]. In agreement with a potential profibrotic role, IL-17 was shown to enhance fibroblast proliferation in humans [15], as well as their production of pro-inflammatory cytokines (MCP-1, IL-6 and IL-8) and matrix metalloproteinases (MMP-1 and MMP-3) [31,32], and ICAM-1 expression [15]. However, Kurasawa and colleagues could not show enhanced type I and type III procollagen mRNA expression in human fibroblasts cultured in the presence of IL-17 [15]. Moreover, Nakashima *et al*. recently provided evidence for an anti-fibrotic effect of IL-17A in human fibroblasts via upregulation of miR-129-5p and downregulation of connective tissue growth factor and α1(I) collagen [33]. In agreement with these findings, we observed that IL-17 decreased alpha-smooth muscle expression induced by transforming growth factor β (TGF-β) in human fibroblasts and that the number of IL-17A + cells in SSc skin correlated inversely with skin fibrosis [34]. Thus, the role of Th17 cells in SSc remains uncertain. The aim of the present study was to investigate whether Th17 cells could promote phenotypic changes in dermal fibroblasts and compare fibroblast responses in healthy and SSc individuals. Our data highlight the direct role of Th17 cells in collagen inhibition accompanied by the simultaneous enhanced production of mediators of inflammation. Furthermore, the data stress the intrinsic resistance of SSc fibroblasts to inhibitory signals delivered by Th17 cells.

## Methods

### Study population

Fourteen SSc individuals (twelve women and two men) presenting at the Rheumatology Unit of the Gaetano Pini Hospital in Milan (Italy) or at the Immunology and Allergy department of the Geneva University Hospital (Switzerland) were prospectively included in the study. All patients met the American Rheumatism Association diagnostic criteria for SSc and were classified according to LeRoy *et al*. [35]. None of the patients were under systemic immunosuppressive therapy apart from a low dose of glucocorticoids (<6 mg per day) at the time of sampling. Eight individuals had limited and six diffuse SSc. A biopsy was performed in the affected skin of the SSc individuals. The control group consisted of eight age and sex matched patients who underwent corrective breast or abdominal surgery at the department of plastic surgery of Clinique de La Tour in Geneva (Switzerland). None of the healthy individuals had dermatological disorders and none were under immunosuppressive agents or glucocorticoids. This study was approved by the ethical committee of the institutions involved (Comité departemental de médicine interne et médicine communautère des Hôpitaux Universitaires de Genève, Geneva, Switzerland; and the Institutional Review Board of the Istituto G. Pini, Milan, Italy) and was conducted according to the Declaration of Helsinki. Written informed consent was obtained from each individual.

### Reagents

Anti-CD3 (clone OKT3) monoclonal antibody (mAb) was from the American Tissue Culture Collection (Manassas, VA, USA); anti-CD4-APC-Cy7, anti-CD45RA-FITC, anti-CCR6-PerCP-Cy5.5, anti-CCR4-PE-Cy7, anti-CXCR3-APC, anti-CD161-APC and anti-CD28 mAbs from BD Biosciences (San Jose, CA, USA); anti-IL-4-APC, anti-IFN-γ-PE-Cy7 and anti-IL-17A-FITC, LEAF irrelevant control mAbs from Biolegend (San Diego, CA, USA); and anti-IL-22-PE, anti-CCR10-PE, recombinant human (rh) IL-23, TGF-β, tumor necrosis factor α (TNF), IL-17 and anti-human IL-17 Ab from R&D Systems (Abingdon, UK). Cytofix/Cytoperm fixation/permeabilization solution kit was from Becton Dickinson (San Diego, CA, USA); Ficoll-Paque Plus from GE Healthcare (Uppsala, Sweden); RPMI 1640, (Dulbecco’s) modified Eagle’s medium ((D)MEM), phosphate buffered saline (PBS), glutamine, penicillin, streptomycin, trypsin and fetal calf serum (FCS) from Gibco (Paisley, UK); phorbol myristate acetate (PMA), ß-mercaptoethanol, α-ketoglutaric acid, β-amino propionitrile, L-ascorbic acid, brefeldin A and nuclear factor-kappaB (NF-κB) peptide inhibitor TPCK from Sigma (St. Louis, MO, USA); rhIL-2 from Biogen (Cambridge, MA, USA); Dynal CD4 Negative Isolation kit from Invitrogen (Oslo, Norway) and phytohemagglutinin (PHA) from EY Laboratories (San Mateo, CA, USA). Radioimmunoassay (RIA) for type I procollagen (PINP-1) was from Orion Diagnostica (Espoo, Finland); and ionomycin, MEK1/2 pharmacological inhibitor U-0126, p38 inhibitor SB203580, JNK inhibitor SP-600125 and PI3K inhibitor LY294002 from Calbiochem (San Diego, CA, USA). TNFα soluble receptor p75 was a kind gift of Dr J Sims, Amgen, Seattle, WA, USA.

### Fibroblast culture

**Figure 1:**
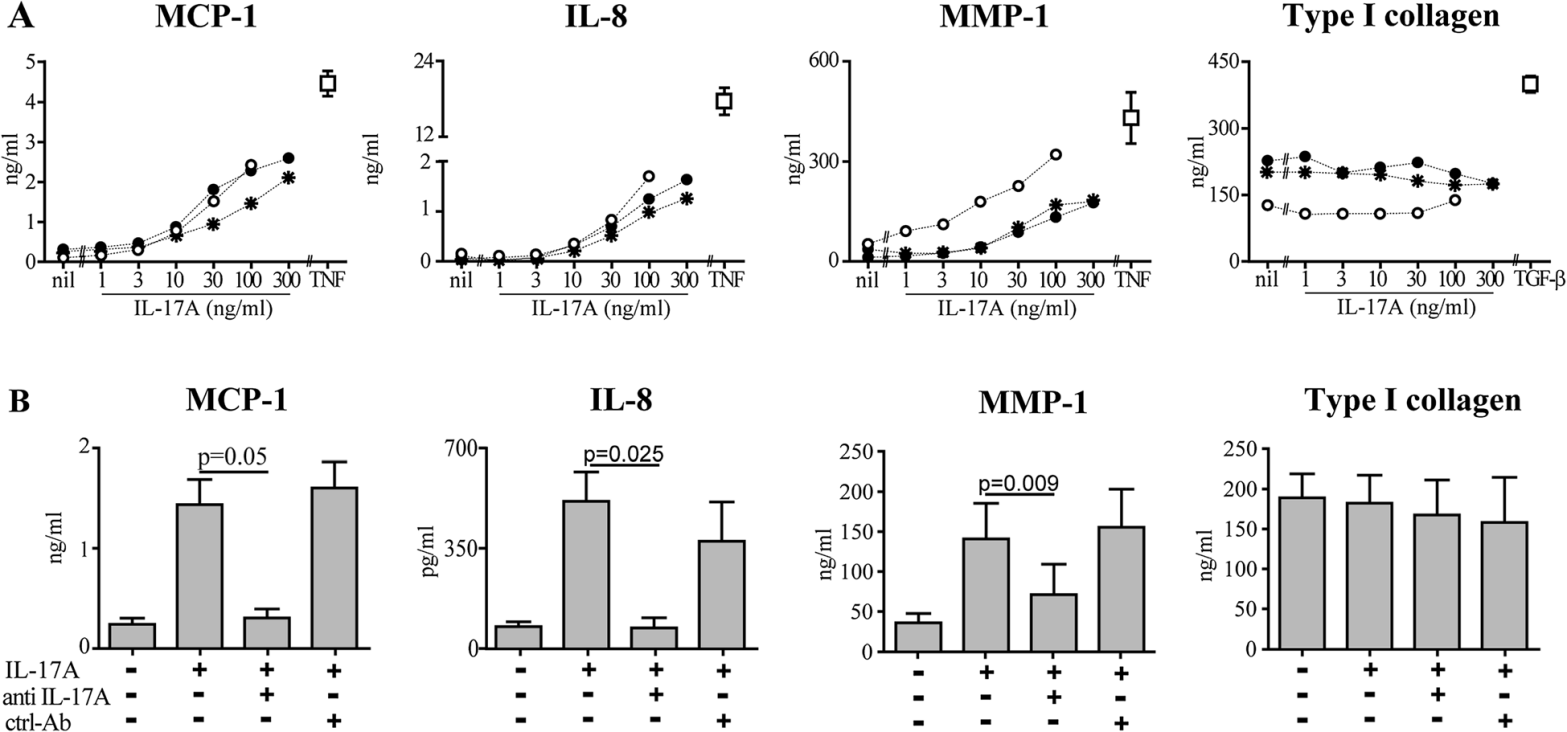
**IL-17A dose-responses in dermal fibroblasts. (A)** Dermal fibroblasts were cultured in the presence of increasing amounts of IL-17A for 48 hours. Shown are MCP-1, IL-8, MMP-1 and type I collagen levels assessed in fibroblast culture supernatants from three distinct HD squares indicates the mean ± SEM of the protein levels in cultures from three HD in the presence of TNF (1 ng/ml) or TGF-β (10 ng/ml) performed in triplicate. **(B)** Bars represent the mean ± SEM of the levels of MCP-1, IL-8, MMP-1 and type I collagen assessed in three distinct HD performed in triplicate. Neutralizing anti-IL-17A and irrelevant control ab (ctrl-Ab) were added one hour before IL-17A. Significant differences were assessed by paired Student’s *t*-test. HD: healthy donors; IL: interleukin; MCP-1: monocyte chemotactic protein-1; MMP-1: matrix metalloprotein-1; SEM: standard error of the mean; TGF: transforming growth factor; TNF: tumor necrosis factor.

Fibroblast cell strains were generated after 0.1% type I collagenase digestion of skin biopsies at 37°C for two hours. Adherent cells were grown in (D)MEM containing 1% nonessential amino acids, 1% L-glutamine, 1% sodium pyruvate, 50 U/ml penicillin, 50 μg/ml streptomycin and 10% FCS. All experiments were performed with fibroblasts at passage 3 to 8. Fibroblasts were seeded at 2 × 10^4^ cells/well in triplicate in 96-well plates for collagen and cytokine assays and at 1 × 10^5^ cells/well in 35-mm tissue culture plates for qPCR and western blot. Cultures were serum-starved overnight and incubated with the indicated reagents in (D)MEM containing 1% FCS, 25 μg/ml L-ascorbic acid, 3.4 μg/ml α-ketoglutaric acid and 50 μg/ml β-amino propionitrile to favor collagen maturation as described [36]. IL-17A was added at 30 ng/ml (which fits in the ascending linear phase of the dose–response curve inducing significant effects, as shown in dose–response experiments in Figure 1A) unless otherwise stated, TGF-β at 10 ng/ml, TNF at 1 or 0.01 ng/ml anti-IL-17A, anti-IFN-γ and irrelevant control mAb at 10 μg/ml (corresponding to a neutralizing antibody to recombinant IL-17A molar ratio of 50:1), anti-TNF (TNF-sRp759) at 10^-8^ M, Th17 supernatants at 1/50 dilution. Supernatants were harvested at 48 hours and frozen until protein determination. Trypsinized cells were snap-frozen in liquid nitrogen and stored at -80°C for total RNA extraction. Alternatively, cells were washed and immediately processed for western blot.

### T cell cloning

CD4 + CD45RA- memory T cells (purity >99%) were isolated from healthy peripheral blood mononuclear cells (PBMC) by negative selection coupling the Dynal CD4 negative Isolation kit with anti-CD45RA mAb. The cells expressing CCR6 + CCR4 + CCR10- and CD161+ were stepwise positively sorted using FACSVantage (Becton Dickinson) to enrich for Th17 cells, resulting in a 7.8-fold enrichment of IL-17-producing CD4+ T cells compared to the parent population. The Th17-enriched cell strains were cloned by limiting dilution in the presence of 0.2 × 10^6^ irradiated (3,500 Rad) allogeneic PBMC and 1 μg/ml PHA in complete RPMI supplemented with 20 U/ml IL-2 and 10 ng/ml of IL-23 as described [37]. The T cell clones obtained were screened for IL-17A, IL-22 and IFN-γ production by intracellular fluorescence-activated cell sorting (FACS) analysis upon 4.5 hour PMA/Inomycin activation in the presence of brefeldin A with specific antibodies using FACSCanto (Becton Dickinson) flow cytometer and FlowJo software 7.5 (Tree Star, Ashland, OR, USA). Selected clones were activated or not by 1 μg/ml coated anti-CD3 and 1 μg/ml soluble CD28 antibodies and supernatants were harvested at 48 hours and frozen for further experiments.

### Chemokine, cytokine and collagen assays

IL-22, MCP-1, MMP-1 and IL-8 were quantified in culture supernatants by ELISA (R&D for IL-22, MCP-1, MMP1; Invitrogen for IL-8). Collagen production was assessed by RIA quantification of PINP (Orion Diagnostica) according to the manufacturer’s instructions. IL-17A, IFN-γ, IL-4 and TNF were quantified by Luminex xMAP^TM^ Technology using multiplex beads immunoassay (Fluorokine MAP Multiplex Human Cytokine Panel, R&D).

### Real-time quantitative PCR

Total RNA was extracted from fibroblasts using an RNAesy micro kit (Qiagen, Hilden, Germany) and cDNA synthesized from 0.25 μg of total RNA using random hexamers and Superscript III reverse transcriptase (Invitrogen, Carlsbad, CA, USA) according to the manufacturer’s instructions. SYBR Green assays were performed on a SDS 7900 HT instrument (Applied Biosystems, Carlsbad, CA, USA). Each reaction was performed in triplicate. Raw cycle threshold (Ct) values obtained with SDS 2.2.2 software (Applied Biosystems) were analyzed and the more stable housekeeping genes (GAPDH (glyceraldehyde-3-phosphate dehydrogenase) and EEF1A1 (eukaryotic translation elongation factor 1 alpha 1)) selected for normalization. All oligonucleotides were obtained from Life Technologies (Carlsbad, CA, USA): CCL2 (F: AACCACAGTTCTACCCCTGGG; R:TAATGATTCTTGCAAAGACCCTCAA), IL8 (F:GCTCTCTTGGCAGCCTTCCT; R:TTAGCACTCCTTGGCAAAACTG), MMP1 (F: GGAGGAAAAGCAGCTCAAGAAC; R:TCCAGGGTGACACCAGTGACT), COL1A1 (F: CCCTCCTGACGCACGG; R:GTGATTGGTGGGATGTCTTCGT), COL1A2 (F:CTGTAAGAAAGGGCCCAGCC; R:GACCCCTTTCTCCACGTGG), MMP2 (F: CTCACAGAACCCTTGGAGCC; R:CCACCAGTGCCCTCTTGAGA), TIMP (F:CGTTATGAGATCAAGATGACCAAGAT; R:CCCCTAAGGCTTGGAACCC), IL-17RA (F:CCTGGAAGTGAAAAATACAGTGATGA; R:AGGCAGGCCATCGGTGT), IL-17RC (F:TGTGCAGTTTGGTCAGTCTGTG; R:GCCTCGAAGCAGTCATATACCAC), EEF1A1 (F: AGCAAAAATGACCCACCAATG; R:GGCCTGGATGGTTCAGGATA) and GAPDH (F: GCACAAGAGGAAGAGAGAGACC; R:AGGGGAGATTCAGTGTGGTG). Expression levels relative to the control condition were calculated using the ΔΔCt method.

### Western blot

Fibroblasts were lysed for 10 minutes on ice in pre-chilled radioimmunoprecipitation assay (RIPA) buffer supplemented with 5 mM ethylenediaminetetraacetic acid (EDTA), 50 mM NaF, 1 mM NasVO4, 100 mM okadaic acid, 1X Complete Protease Inhibitor Cocktail (Roche, Basel, Switzerland) and 0.2 mM phenylmethylsulfonyl fluoride (PMFS). Protein extracts were clarified by centrifugation and stored at -20°C until use. For western blot, 30 μg of total protein extract were separated in 10% SDS-PAGE, under reducing conditions, and electroblotted onto nitrocellulose membranes (Amersham^TM^ Hybond^TM^-ECL, GE Healthcare Zurich, Switzerland). Blots were incubated with antibodies against phospho-extracellular signal-regulated kinase (ERK)1/2 (Thr202/Tyr204), phospho-p38 (Thr180/Tyr182), phospho-c-Jun (Ser73), phospho-Smad2 (Ser465/467), IκB-α, phospho-IκB-α(Ser32), phospho-AKT (Ser473) (Cell Signaling, Danvers, MA, USA), phospho-c-Jun N-terminal kinases (JNK) (G-7) (Santa Cruz Biotechnology, Inc., Santa Cruz, CA, USA) and β-tubulin (Sigma). Horseradish peroxidase-conjugated antisera were used to reveal primary binding, followed by detection by an ECL system (GE Healthcare). Quantification analysis was performed with ImageJ software (http://rsbweb.nih.gov/ij) and values were normalized to β-tubulin.

### Statistical analysis

Statistical analysis was performed with GraphPad Prism version 4.00 (Graphpad Software, La Jolla, CA, USA). Significant difference between samples was computed using Student’s *t*-test for paired or unpaired samples according to the experimental design. The Wilcoxon signed-rank test was used to compare fold changes in protein or mRNA levels relative to the control condition. A *P* value <0.05 was considered statistically significant.

## Results

### IL-17A enhances MCP-1, IL-8 and MMP-1 but not type I collagen production in HD and SSc dermal fibroblasts

**Figure 2:**
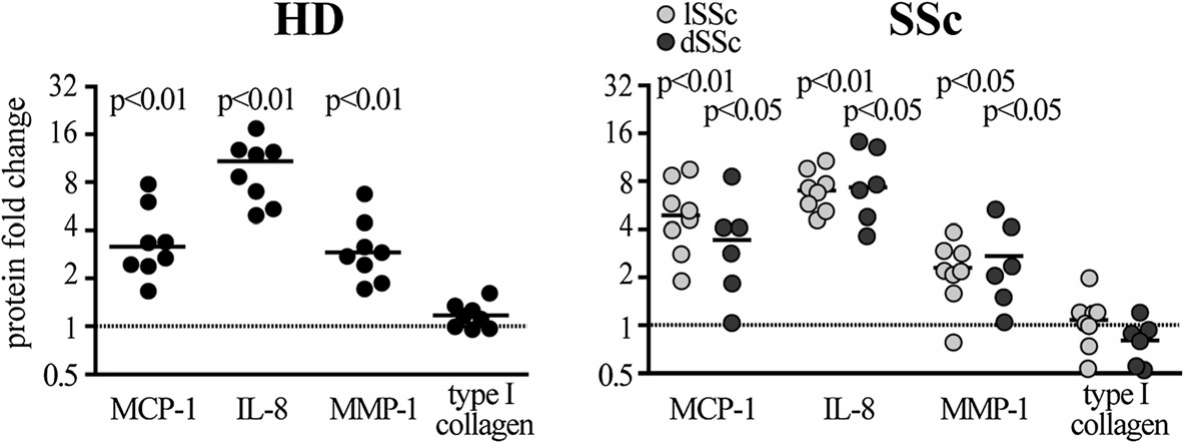
**IL-17A enhances MCP-1, IL-8 and MMP-1 but not collagen production in HD and SSc fibroblasts.** Dermal fibroblasts were cultured in the presence of 30 ng/ml of IL-17A. Protein levels are expressed as fold change relative to control condition (medium with no IL-17A, dotted line) assessed in 48-hour culture supernatants by ELISA (MCP-1, IL-8, MMP-1) and RIA (collagen). Each symbol represents a distinct individual and the horizontal lines depict the median. Significant differences relative to the control condition were assessed by the Wilcoxon signed-rank test. ELISA: enzyme-linked immunosorbent assay; IL: interleukin; MCP-1: monocyte chemotactic protein-1; MMP-1: matrix metalloprotein-1; RIA : radio-immune assay.

**Figure 3:**
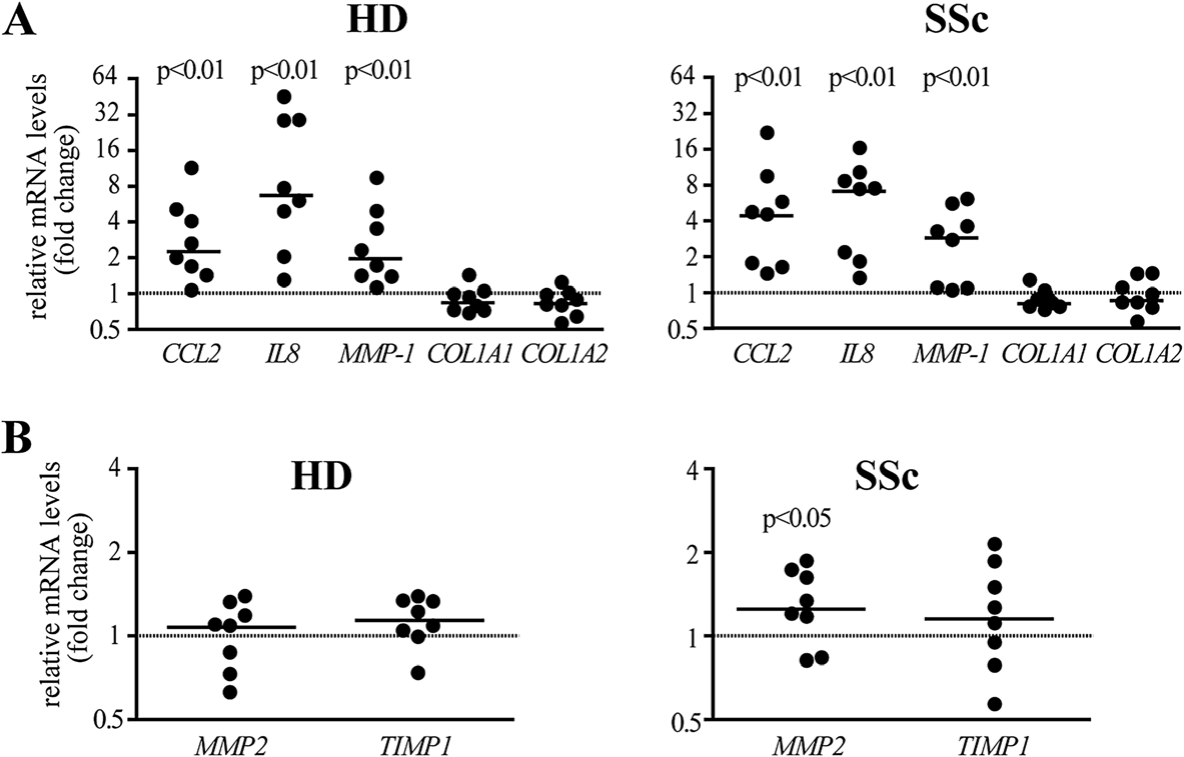
**IL-17A enchances the mRNA levels of MCP-1, IL-8 and MMP-1 with no effects of COL1A1 and COL1A2 in HD and SSc fibroblasts. (A-B)** Steady-state mRNA levels were quantified by real-time PCR after 24 hours of culture. Expression levels were normalized against the geometric mean of two house-keeping genes (GADPH, EEF1A1). In all panels, the results are expressed as fold change relative to the control condition (medium with no IL-17A, dotted line) Each symbol represents a distinct individual and the horizontal lines depict the median. Significant differences relative to the control condition were assessed by the Wilcoxon signed-rank test. Fold increase induced by positive control TNF: 28 ± 38 (*CCL2*), 448 ± 317 (*IL8*), 18 ± 22 (*MMP1*), 1.5 ± 0.5 (*MMP2*), 1.6 ± 0.7 (*TIMP1*) and TGF-β: 3.8 ± 2.1 (*COL1A1*), 1.8 ± 0.6 (*COL1A2*). CCL: CC-chemokine ligand; COL: collagen; EEF1A1: eukaryotic elongation factor 1 alpha 1; GADPH: glyceraldehyde 3 phosphate dehydrogenase; HD: healthy donors; IL: interleukin; MCP-1: monocyte chemotactic protein-1; MMP-1: matrix metalloprotein-1; PCR: polymerase chain reaction; SSc: systemic sclerosis; TGF: transforming growth factor; TIMP: tissue inhibitor of matrix metalloproteinase; TNF: tumor necrosis factor.

Several lines of evidence indicate that Th17 cells and their hallmark cytokine IL-17A are increased in SSc [15-21]. We therefore assessed whether IL-17A can affect the capacity of dermal fibroblasts from SSc and HD to produce inflammatory cytokines and ECM components known to be upregulated in SSc. Expanding previous observations [15,31,32], IL-17A enhanced the production of MCP-1, IL-8 and MMP-1 in a dose-dependent manner (Figure 1A). Neutralization of IL-17A completely abrogated the responses induced by IL-17A, thus confirming the specificity of our findings (Figure 1B). MCP-1, IL-8 and MMP-1 responses were similar in SSc and HD fibroblasts at both the protein and mRNA levels (Figures 2 and 3A). Of interest, IL-17A, even at high doses, did not affect type I collagen production, which production was enhanced in response to TGF-β, used as positive control (Figures 1A and 3A). With respect to the cohort analyzed, no difference in MCP-1, MMP-1, IL-8 and type I collagen production was observed between limited systemic sclerosis (lSSc) and diffuse systemic sclerosis (dSSc) individuals (Figure 2).

Consistently, IL-17A did not modify *COL1A1* and *COL1A2* mRNA levels both in SSc and HD fibroblasts (Figure 3A). Finally, IL-17A did not affect the mRNA levels of TIMP-1, and slightly, but significantly, enhanced *MMP2* mRNA in SSc but not HD fibroblasts (Figure 3B).

Together, our findings demonstrate that IL-17A directly contributes to fibroblast inflammatory responses by enhancing MCP-1 and IL-8 production, and simultaneously impacts on ECM turnover by favoring MMP-1 rather than type I collagen production.

### IL-17A effects on pro-inflammatory chemokines (MCP-1, IL-8) and MMP-1 are mediated by distinct signaling pathways

**Figure 4:**
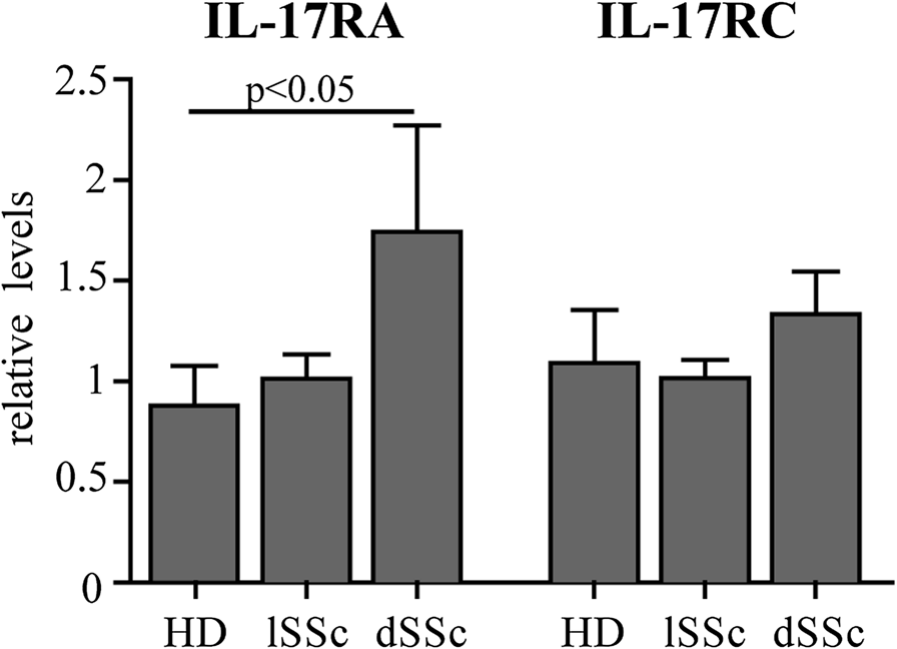
**mRNA expression levels of IL-17RA and IL-17RC in HD and SSc fibroblasts.** Bars represent the mean ± SD of the mRNA levels at basal conditions of IL-17RA and IL-17RC normalized to the geometric mean of two house-keeping genes (GADPH, EEF1A1) in eight HD, eight lSSc and six dSSc fibroblasts. Significant differences were assessed by *t*-test. dSSc: diffuse systemic sclerosis; EEF1A1: eukaryotic elongation factor 1 alpha 1; GADPH: glyceraldehyde 3 phosphate dehydrogenase; HD: healthy donors; IL: interleukin; lSSc: limited systemic sclerosis.

**Figure 5:**
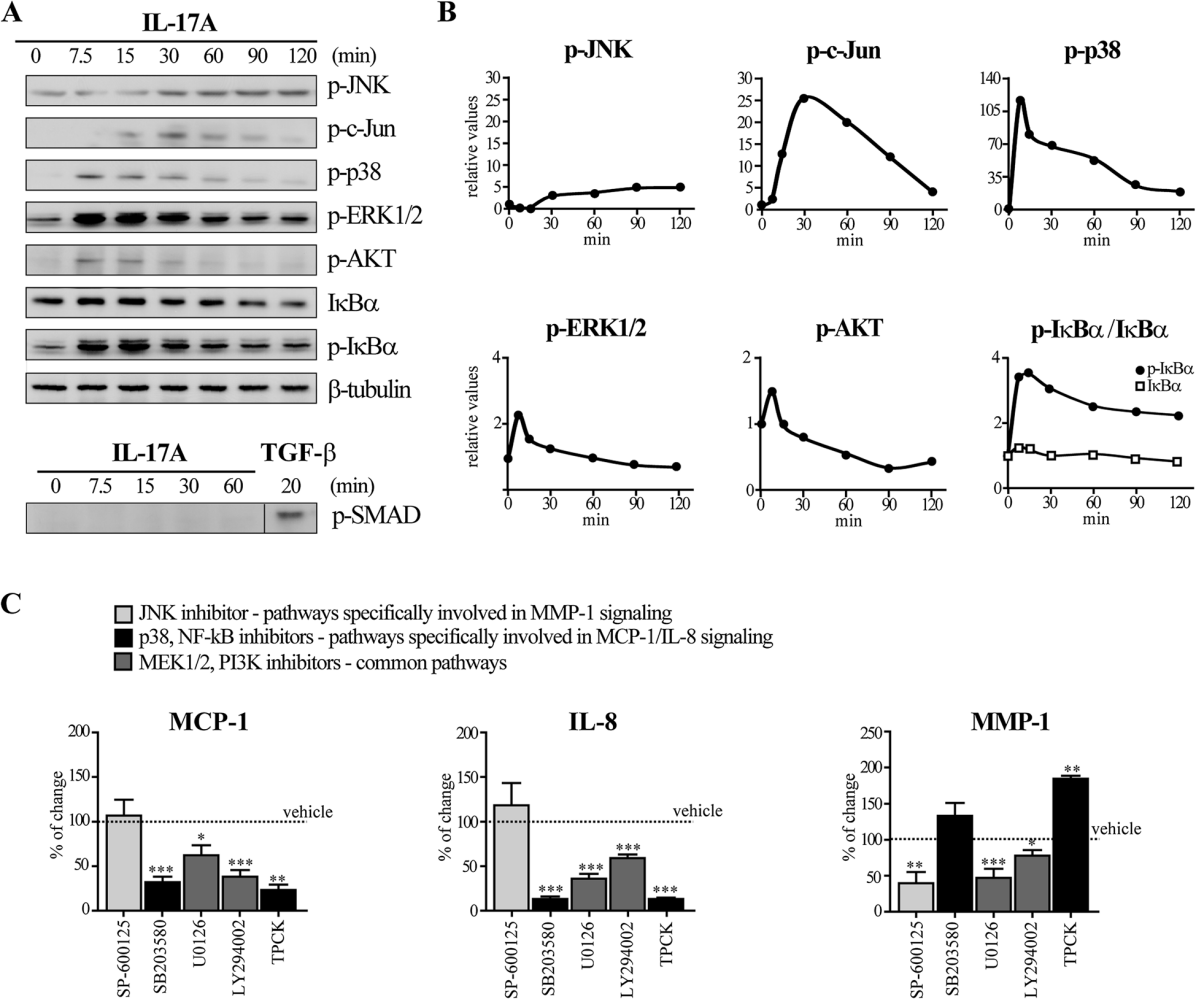
**IL-17A induces the production of pro-inflammatory chemokines and MMPs by triggering distinct signaling pathways. (A)** Detection of signaling proteins following SDS-PAGE and immunoblotting in lysates of dermal fibroblasts from one representative HD of three cultured in the presence of IL-17A for the indicated time-points. **(B)** Densitometric analysis of the signaling proteins normalized to β-tubulin levels. Data are expressed as the ratio of the normalized values relative to time = 0 minutes. One representative experiment of three is shown. **(C)** Protein levels were assessed in 48-hour culture supernatants by ELISA. Bars represent the mean ± SEM from six distinct HD performed in triplicate relative to the control condition (vehicle). IL-17A (30 ng/ml) was added in all conditions. When used, SP-600125 (JNK inhibitor, 10 μM), SB203580 (p38 inhibitor, 20 μM), U0126 (MEK1/2 inhibitor, 20 μM), LY294002 (PI3K inhibitor, 10 μM) and TPCK (NF-κB inhibitor, 5 μM) were added one hour before IL-17A. Significant differences versus vehicle were assessed by Student’s *t*-test: * = *P* <0.05, ** = *P* <0.01, *** = *P* <0.001. Light gray, black and dark gray bars depict pathways involved in MMP-1 signaling, MCP-1/IL-8 signaling and common pathways, respectively. ELISA: enzyme-linked immunosorbent assay; HD: healthy donors; IL: interleukin; JNK: c-jun N-terminal kinase; MCP: monocyte chemotactic protein; MEK: mitogen-activated protein kinase; MMP: matrix metalloproteinase; NF-kB: nuclear factor kappa B; PI3K: phosphoinositol 3 kinase; SDS-PAGE: Sodium Dodecyl Sulphate - PolyAcrylamide Gel Electrophoresis; SEM: standard error of the mean.

IL-17A binds to and signals via a heterodimeric IL-17 receptor composed of the IL-17RA and IL-17RC subunits. When compared to normal fibrobalsts, only dSSc but not lSSc fibroblasts showed higher IL-17RA mRNA relative levels (Figure 4). The relative levels of IL-17RC mRNA were similar across the three study groups (Figure 4). IL-17A activated several intracellular signaling pathways including c-Jun/JNK, ERK 1/2, p38 and protein kinase B (AKT) as demonstrated by time-dependant modifications in their phosphorylation levels (Figure 5A and B). In addition, IL-17A induced the phosphorylation of the NF-κB inhibitor protein IκBα, while it did not trigger Smad2-phosphorylation, which was high in response to the positive control, TGF-β (Figure 5A and B). The production of MCP-1, IL-8 and MMP-1 was reduced in the presence of the specific MAP Kinase Kinase (MEK)1/2 (upstream of ERK1/2) inhibitor U0126 and PI3K (upstream of AKT) inhibitor LY294002, suggesting a wide involvement of these pathways in transducing IL-17A signals (Figure 5C, dark gray bars). Interestingly, the increased production of the pro-inflammatory chemokines MCP-1 and IL-8, but not that of MMP-1 was abrogated by the p38 inhibitor SB203580 and the NF-κB inhibitor TPCK (Figure 5C, black bars). In contrast, MMP-1, but not pro-inflammatory chemokine production was strongly reduced when JNK was inhibited by SP-600125 (Figure 5C, light gray bars). Thus, our data indicate that IL-17A exploits distinct signaling pathways to favor the production of pro-inflammatory chemokines (p38 and NF-κB dependent) and MMP-1 (JNK dependent).

### Th17 clones enhance MCP-1, IL-8 and MMP-1 and decrease type I collagen production to different extents in HD and SSc fibroblasts

**Figure 6:**
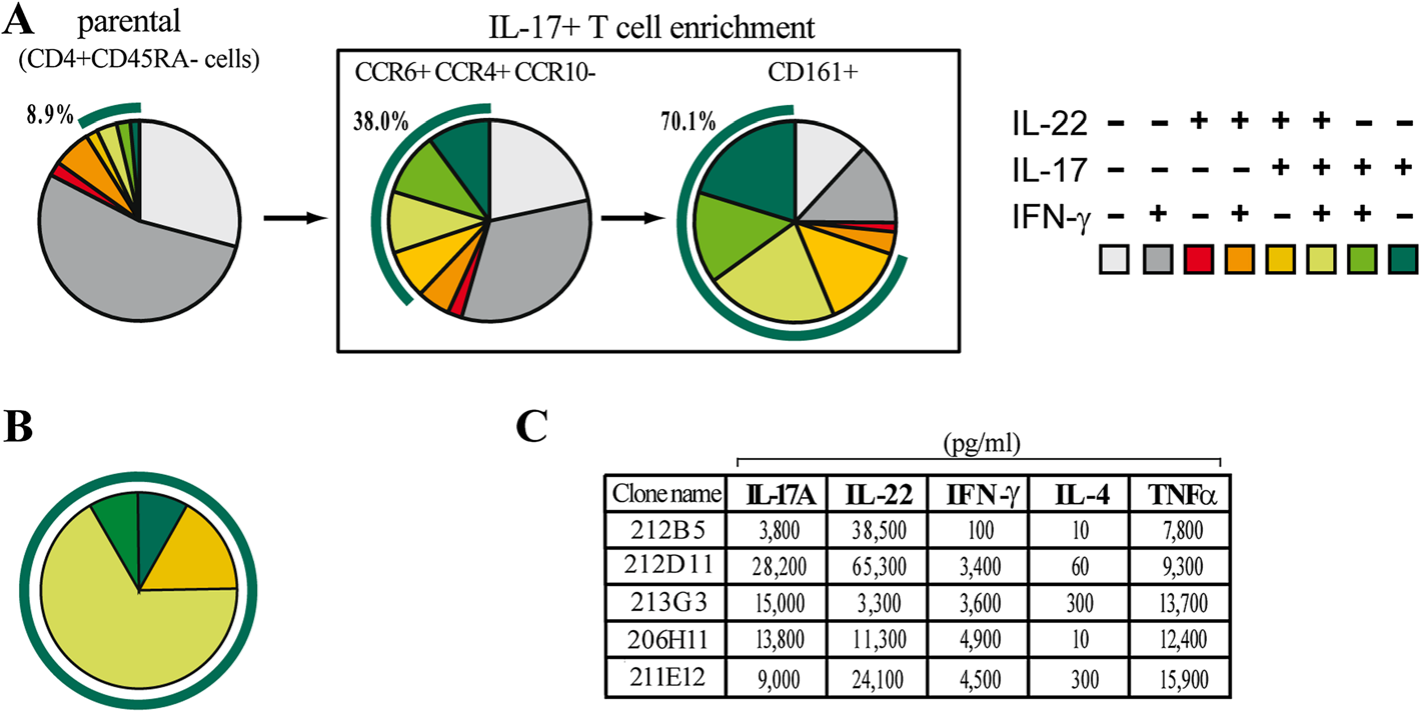
**Th17 cell clone generation and characterization. (A**, **B)** Boolean gating analysis showing all combinations of IL-17A, IL-22 and IFN-γ production by CD4 + CD45RA- memory T cells before and after stepwise enrichment based on CCR6 + CCR4 + CCR10- and CD161+ surface expression **(A)**, and in the 20 T cell clones expanded after enrichment **(B)**. Intracellular cytokine staining was performed after PMA and ionomycin stimulation. Color codes are shown in the right panel of A. The green circular segment indicates the total number of IL-17A-producing T cells after each step of enrichment. **(C)** T cell cytokine levels were measured in supernatants of five of the twenty activated Th17 cell clones by ELISA and bead immunoassay. CCR: CC-chemokine receptor; CD: cluster of differentiation; ELISA: enzyme immunosorbent assay; IFN: interferon; IL: interleukin; PMA: phorbol myristate acetate.

**Figure 7:**
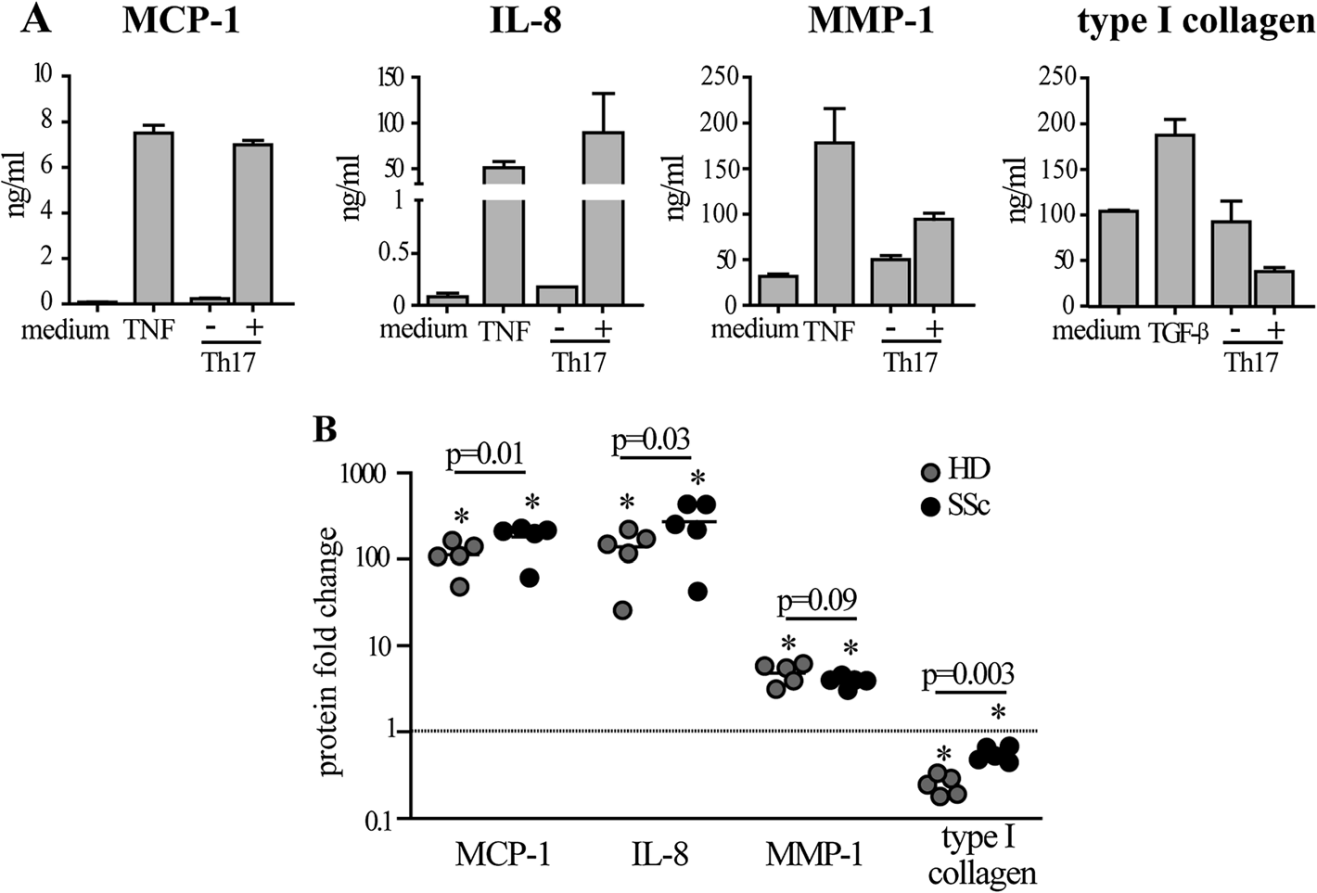
**Th17 clones enhance MCP-1, IL-8 and MMP-1 and decrease type I collagen production by fibroblasts. (A)** MCP-1, IL-8, MMP-1 and type I collagen production by HD fibroblasts cultured for 48 hours in the presence of supernatants of a prototypical Th17 clone supernatant before (-) or after (+) CD3/CD28 activation. TNF (1 ng/ml) and TGF-β (10 ng/ml) were used as positive controls. **(B)** Fold change relative to the control condition (dotted line) of MCP-1, IL-8, MMP-1 and type I collagen production by HD (gray) and SSc (black) fibroblasts cultured for 48 hours in the presence of supernatants from five distinct, activated Th17 clones. Each symbol represents an individual clone and the solid horizontal lines depict the median. Significant differences relative to the control condition were assessed by the Wilcoxon signed-rank test: * = *P* <0.05. Significant differences between HD and SSc are shown. CD: cluster of differentiation; HD: healthy donors; IL: interleukin; MCP-1: monocyte chemotactic protein-1; MMP-1: matrix metalloprotein-1; SSc: systemic sclerosis.

We then investigated whether the effects induced by Th17 cells on dermal fibroblasts were similar to that induced by IL-17A. To this aim we generated human Th17 cell clones. Since the frequency of Th17 cells in the PBMC is very low (below 1%), we adopted a strategy to generate Th17 clones by a stepwise approach. In a prototypical experiment, we found that 8.9% of the CD4 + CD45RA- peripheral blood T cells were producing IL-17A (1.5% were producing IL-17A alone and 7.4% IL-17A in conjunction with IL-22 and IFN-γ). The frequency of IL-17A-producing T cells was enriched up to 38.0% upon positive sorting of CCR4 + CCR6+ cells and to a further 70.1% after positive sorting of CD161+ cells (Figure 6A). This IL-17A + enriched T cell population was then cloned by limiting dilution. Several of the 20 screened clones (of 53 generated) produced high levels of IL-17A with variable levels of IL-22 and IFN-γ, thus being Th17 or Th17/Th1 cells [38] (Figure 6B). The supernatants of five distinct, representative clones were generated for further experiments. Of note, substantial amounts of TNF were produced by all clones (Figure 6C). All supernatants from activated, but not from resting, Th17 cell clones strongly induced MCP-1, IL-8 and MMP-1 and inhibited type I collagen production by both HD and SSc fibroblasts (Figure 7A and B). However, the production of MCP-1 and IL-8 was higher, while collagen inhibition was lower in SSc compared to HD fibroblasts (Figure 7B). When compared to recombinant IL-17A, Th17 cell clone supernatants induced higher levels of pro-inflammatory chemokines (MCP-1 and IL-8) and similar levels of MMP-1. Of note and different from IL-17A, Th17 clones strongly inhibited type I collagen production (Figures 2, 3 and 7). Thus, quantitative as well as qualitative differences were observed in fibroblast responses when stimulated by Th17 cell supernatants compared to recombinant IL-17A.

### Th17 cell supernatant effects are mainly mediated by IL-17A, TNF and, in part, IFN-γ

**Figure 8:**
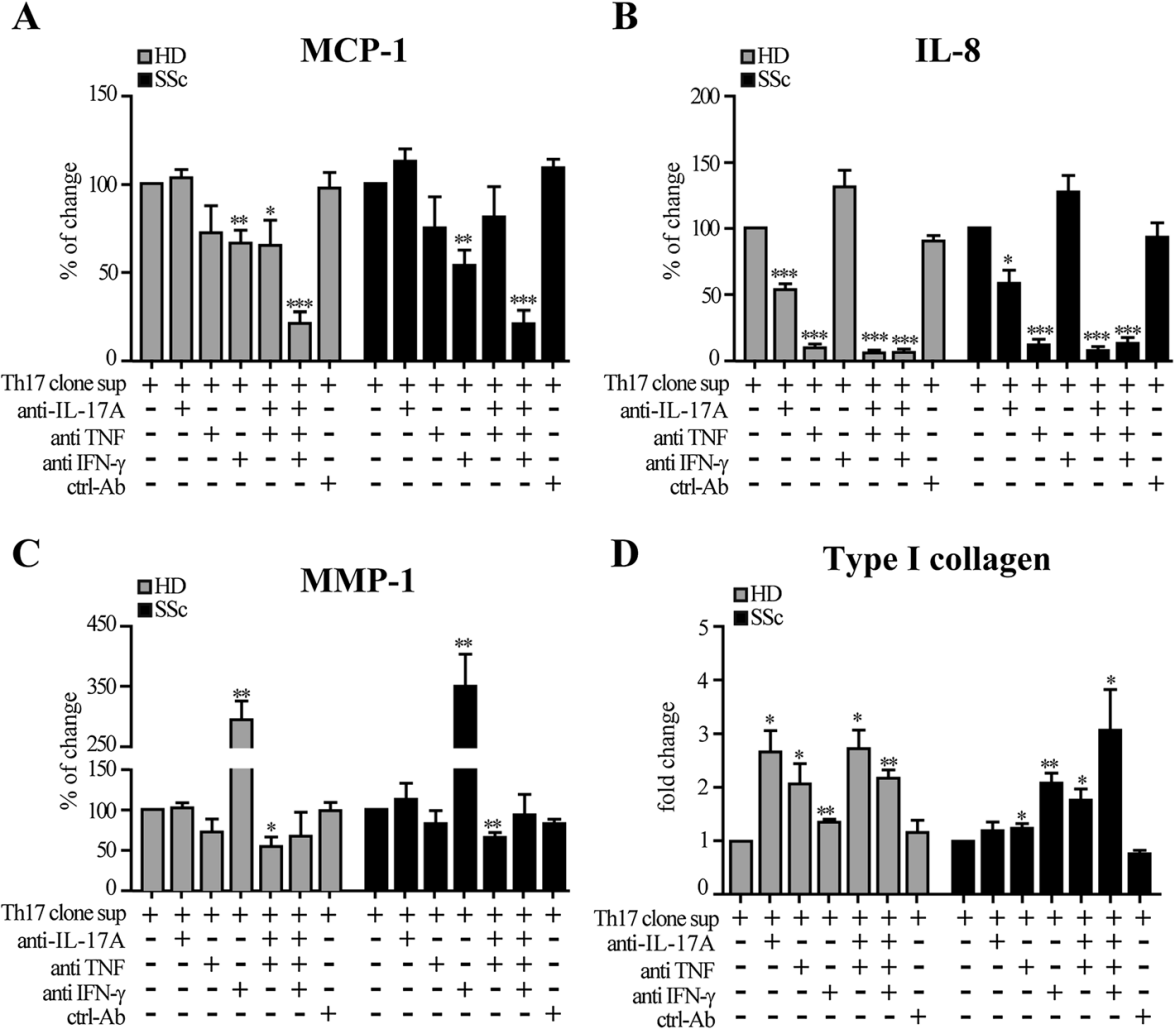
**Th17 clone effects on fibroblasts are mediated in part by IL-17A, TNF and IFNγ.** MCP-1 **(A)**, IL-8 **(B)**, MMP-1**(C)** and type I collagen **(D)** production by HD (gray) and SSc (black) fibroblasts cultured for 48 hours in the presence of supernatants of five distinct, activated Th17 clones. Bars represent the mean ± SEM of triplicate experiments. All data are expressed as the percentage of change relative to the condition in which the Th17 clone supernatants were added in the absence of blocking reagents (namely 'Th17 clone sup’). Anti-IL-17A (10 μg/ml), anti-IFN-γ (10 μg/ml), TNF-sRp75 (10^-8^ M) and ctrl Ab (10 μg/ml) were added one hour before the beginning of the culture. Shown are significant differences relative to the 'Th17 clone sup’ condition (* = *P* <0.05; ** = *P* <0.01) assessed by paired *t*-test. ctrl: control; : healthy donors; IL: interleukin; IFN: interferon; MCP-1: monocyte chemotactic protein-1; MMP-1: matrix metalloprotein-1; PCR: polymerase chain reaction; SSc: systemic sclerosis; sup: supernatant; TNF: tumor necrosis factor; TNF-sRp75: tumor necrosis factor soluble receptor protein 75.

**Figure 9:**
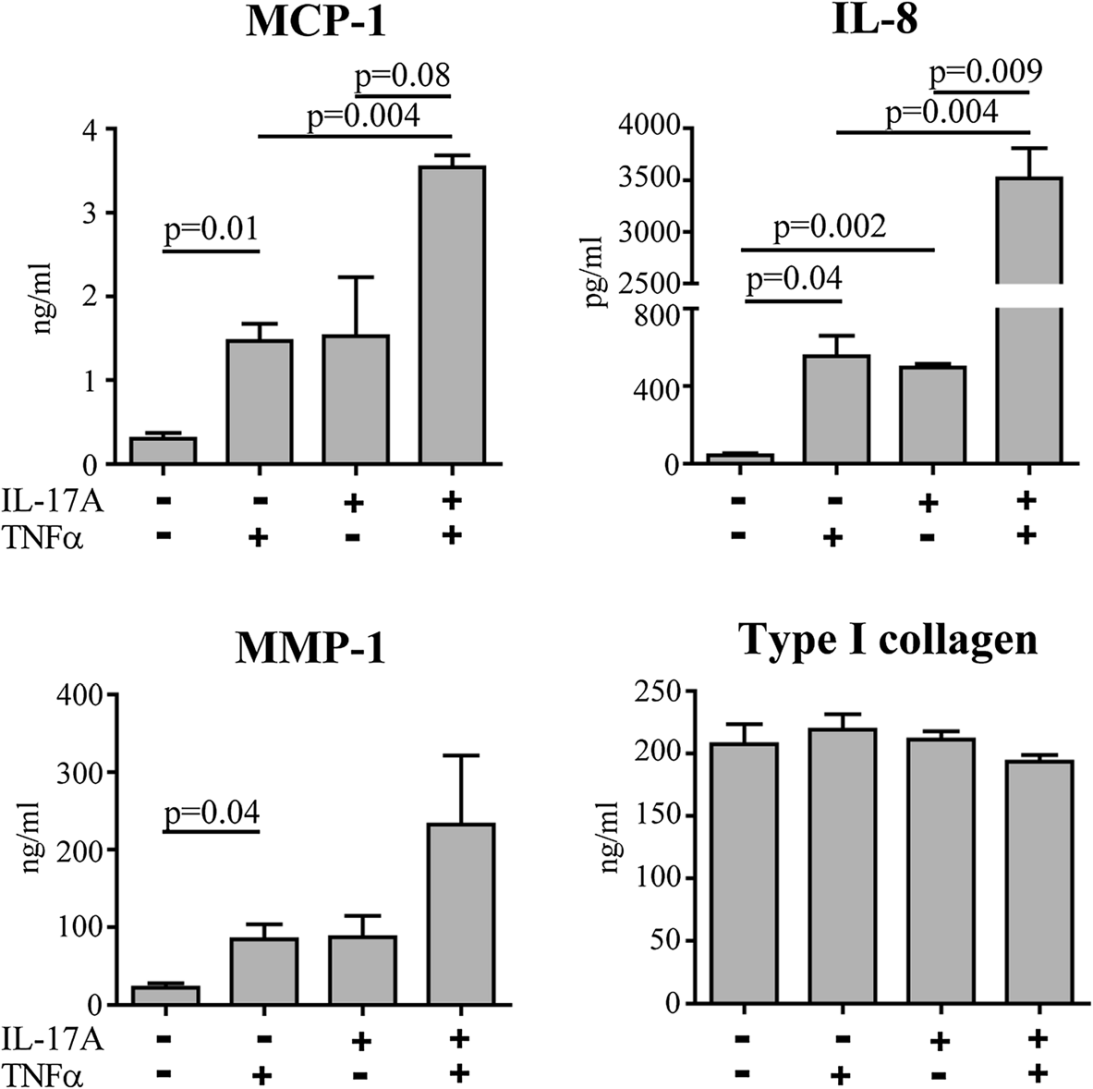
**IL-17A synergizes with TNF to induce the production of MCP-1, IL-8 and MMP-1 by dermal fibroblasts.** Bars represent the mean ± SEM of the levels of MCP-1, IL-8, MMP-1 and type I collagen assessed in the supernatants of dermal fibroblasts from three HD cultured in triplicate for 48 hours in the presence or absence of IL-17A (30 ng/ml) and TNF (0.01 ng/ml). Statistically significant differences were assessed by paired *t*-test. HD: healthy donors; IL: interleukin; MCP-1: monocyte chemotactic protein-1; MMP-1: matrix metalloprotein-1; SEM: standard error of the mean; TGF: transforming growth factor; TNF: tumor necrosis factor.

As mentioned above and shown in Figure 6C, Th17 cell supernatants contained several cytokines in addition to IL-17A. We, therefore, assessed to which extent the effects observed in fibroblasts were mediated by IL-17A. IL-17A blockade significantly decreased the production of IL-8, but not that of MCP-1 and MMP-1, induced by five different Th17 cell clones by both HD and SSc fibroblasts (Figure 8A, B and C). Similar effects were observed upon TNF blockade (Figure 8A, B and C). The simultaneous blockade of IL-17A and TNF resulted in a maximal inhibition of IL-8 and MMP-1 (Figure 8A, B and C). In keeping with these observations, recombinant IL-17A synergized with recombinant TNF in enhancing IL-8 and MMP-1 production when added to HD fibroblasts (Figure 9). Of interest, IFN-γ blockade in the same supernatants resulted in slightly decreased MCP-1 and strongly increased MMP-1 with no effect on IL-8 production (Figure 8A, B, C). Maximal inhibition of MCP-1 was observed when IL-17A, TNF and IFN-γ were simultaneously blocked both in SSc and HD fibroblasts (Figure 8A, B, C). Interestingly, IL-17A or TNF blockade partially reverted the inhibition of type I collagen production induced by the Th17 cell clones in HD and only minimally in SSc fibroblasts (Figure 8D). Conversely, neutralization of IFN-γ resulted in a reversion of collagen inhibition particularly in SSc and only minimally in HD fibroblasts, again stressing phenotypic differences intrinsic in SSc fibroblasts. Of major interest, the joint blockade of IL-17A and TNF or IL-17A, TNF and IFN-γ resulted in the complete reversal of collagen inhibition induced by Th17 clones mostly in SSc fibroblasts (Figure 8D).

## Discussion

In the present report, we show that Th17 cells elicit MCP-1, IL-8 and MMP-1 responses while simultaneously inhibiting type I collagen production in healthy and SSc dermal fibroblasts. Our data are consistent with a model in which Th17 cells participate in inflammatory events but not directly in enhanced collagen deposition. In this perspective, Th17 cells may be seen as cells with an important role in limiting the development of fibrosis. In line with our data, a recent work by Nakashima *et al*. indicated that IL-17A may have direct anti-fibrotic effects in human normal fibroblasts via upregulation of miR-129-5p and downregulation of connective tissue growth factor and type I collagen [15]. According to these authors, SSc fibroblasts may escape the negative control of IL-17A because of a reduced expression of the IL-17RA [15]. In our experimental settings, diffuse SSc fibroblasts expressed increased IL-17RA mRNA levels but, in partial agreement with Nakashima *et al*., we observed that collagen production by SSc fibroblasts was more resistant to inhibition by Th17 cells. Additional *in vivo* evidence consistent with this model was obtained when we studied the number of IL-17A + cells in the skin of SSc individuals and found that the total skin thickness score was higher when IL-17A + dermal cells were less numerous [34]. Of interest, Th17 cell numbers can be increased both *in vitro* and *in vivo* by iloprost, a PGI_2_ analog used in the clinical management of SSc digital ulcers, which may have beneficial effects on the disease course [39]. These data and our model are distinctly different from data and conclusions generated in rodents, in which IL-17 was shown to favor *in vivo* collagen deposition in models of bleomycin-induced skin as well as lung fibrosis [29,30,40]. Furthermore, in the thigh skin of mice lacking IL-17 the spontaneous fibrotic skin was reduced [30], and finally IL-17 neutralization decreased lung inflammation and fibrosis induced by silica [41]. The discrepancy between studies in humans and mice stresses species-specific differences in the responses induced by IL-17, as thoroughly discussed recently [8].

Our data clearly show that IL-17A directly promotes the production of pro-inflammatory mediators and MMP-1 by dermal fibroblasts from healthy and SSc individuals. Within the limits of the cohort investigated in this study, no differences were observed between limited and diffuse SSc individuals in this respect. These effects were largely amplified when supernatants from Th17 cell clones, producing high levels of IL-17, were assessed. Neutralizing experiments confirmed a critical role for IL-17A, at least in the case of IL-8, and revealed additive/synergic effects of IL-17 and TNF. Along this line of evidence, IL-17 was shown to enhance TNF-induced synthesis of IL-1, IL-6 and IL-8 by normal skin fibroblasts and osteoarthritis fibroblast-like synoviocytes [42]. MCP-1 and IL-8 are increased in skin and serum of SSc patients [5,43] and reported to be critical in mediating lung and dermal fibrosis in bleomycin-treated mice [6,7]. However, whether these mediators have direct pro-fibrotic activities in humans is controversial. An increase in α1(I) collagen mRNA was reported by northern blot hybridization in human dermal fibroblasts activated by MCP-1 [5], while later reports could not confirm these findings [44]. Similarly, MCP-1 was reported to increase the expression of MMP-1 and MMP-2, critical matrix degrading enzymes, but also the levels of their inhibitor TIMP-1 [45]. The role of these mediators in tissue fibrosis observed in mice may be related more to chemoattractant and angiogenetic properties than to a direct pro-fibrotic activity on fibroblasts or to its role in favoring priming of Th2 cells [46,47].

We found that IL-17A enhanced MMP-1 production in dermal fibroblasts, as previously reported in human cardiac fibroblasts and fibroblast-like synoviocytes [48-51]. MMPs participate in tissue remodeling, directly acting on ECM but also modulating the activity of many important mediators regulating matrix deposition [52]. Despite its role as a degrading enzyme, MMP-1 levels have been paradoxically shown to be highly increased in human lung fibrosis [53], and variably reported to be increased, unchanged or decreased in SSc [54-57]. Thus, the exact role of MMP-1 in the development of fibrosis remains to be established.

We showed that IL-17A induced the production of pro-inflammatory chemokines preferentially via NF-κB and p38 signaling pathways, while inducing MMP-1 via JNK. Consistent with our data, IL-17 was previously shown to promote IL-6/IL-8 production via NF-κB/Akt and NF-κB/MAPK pathways in rheumatoid arthritis synovial fibroblasts and colonic myofibroblasts, respectively [58,59] and in partial agreement with our findings, IL-17 induced MMP-1 production via activation of c-Fos/c-Jun AP1 and NF-κB in addition to MAPK signaling in cardiac fibroblasts [49].

Th17 cell clones were obtained after enrichment of cells expressing the chemokine receptor CCR6 and CCR4 in the absence of CCR10 [26,27] and the lectin receptor CD161 [28]. By applying this strategy, we obtained more than 70% of cells producing IL-17A. Compared to the expected numbers, the cloning procedure resulted in a slight enrichment of clones co-producing IL-17 and IFN-γ (Th1/Th17 cells), suggesting a relationship between the Th1 and Th17 differentiation programs. In line with these results, a functional plasticity connecting Th1 and Th17 cells was recently reported both *in vitro* and *in vivo*[28,38,60], although IL-17+/IFN-γ + cells were shown to have a transcription profile closer to Th17 than to Th1 cells [38].

Of note, SSc fibroblasts were more prone to produce pro-inflammatory mediators (MCP-1, IL-8) and less sensitive to collagen inhibition when cultured in the presence of Th17 cell clone supernatants than their healthy counterpart. This suggests that SSc fibroblasts may escape or limit the anti-fibrotic effects induced by Th17 cells, and further stresses the existence of intrinsic differences between normal and SSc fibroblasts. In this context, it is worth noting that the inhibition of type I collagen production induced by the Th17 clone supernatants was partially reversed by blockade of IL-17 or TNF mainly in HD but not SSc fibroblasts while IFN-γ neutralization had opposite effects. Again, the joint blockade of IL-17, TNF and IFN-γ resulted in maximal effects, specifically in SSc but not HD fibroblasts. In agreement with previous evidence [36,61], the present data strongly suggest that, compared to normal fibroblasts, SSc fibroblasts are more resistant to inhibitory mediators present in the Th17 cell clone supernatants.

In conclusion, our data are consistent with a model in which Th17 cells may participate in enhancing inflammation while simultaneously limiting fibrosis. It is worth noting that the contribution of Th17 cells to inflammatory conditions remains in many instances a matter of debate. As an example, the role of IL-17 in the initiation, progression and stabilization of atherosclerosis is currently controversially interpreted with evidence in favor of its proatherogenic potential and evidence in favor of its atheroprotective role [62]. Our findings stress for the first time the concomitant dual role of Th17 cells in the context of matrix deposition and may provide the functional basis for novel approaches to harness fibrotic diseases.

## Conclusions

Th17 cells enhance *in vitro* fibroblast inflammatory responses while simultaneously inhibiting collagen production with a mechanism partially dependent on IL-17, TNF and IFN-γ. SSc fibroblasts are, however, intrinsically resistant to collagen inhibition induced by Th17 cells. Thus, the increased Th17 cell counts observed in SSc might be considered a manifestation of autoimmunity not mechanistically linked to fibrosis.

## Abbreviations

CCR: CC chemokine receptor; Ct: Cycle threshold; dSSc: Diffuse systemic sclerosis; ECM: Extracellular matrix; ELISA: Enzyme-linked immunosorbent assay; ERK: Extracellular signal-regulated kinase; FCS: Fetal calf serum; HD: Healthy donor; IFN-γ: Interferon gamma; IL: Interleukin; JNK: c-Jun N-terminal kinases; lSSc: Limited systemic sclerosis; mAb: Monoclonal antibody; MCP: Monocytes chemoattractant protein; MEK: MAP kinase kinase; MMP: Matrix metalloproteinase; NF-κB: Nuclear factor-kappaB; PBMC: Peripheral blood mononuclear cell; PHA: Phytohemagglutin; RIA: Radioimmunoassay; SSc: Systemic sclerosis; TGF: Transforming growth factor; Th: T helper; TIMP: Tissue inhibitor of metalloproteinase; TNF: Tumor necrosis factor.

## Competing interests

The authors declare that they have no competing interests.

## Authors’ contributions

NCB, EM and MET conceived and designed the experiments, and acquired, analyzed and interpreted the data. ER acquired the data and designed the experiments. CC conceived and designed research, and interpreted the data. PLM conceived and designed research. NCB and CC drafted the manuscript. All authors read and approved the final manuscript.
